# Molecular Identification of *Enterocytozoon bieneusi* Isolates from Nigerian Children

**DOI:** 10.1155/2011/129542

**Published:** 2011-11-03

**Authors:** Adekunle Bamidele Ayinmode, Oladele Teslim Ojuromi, Lihua Xiao

**Affiliations:** ^1^Department of Veterinary Microbiology and Parasitology, Faculty of Veterinary Medicine, University of Ibadan, Ibadan, Nigeria; ^2^Division of Foodborne, Waterborne, and Environmental Diseases, Centers for Disease Control and Prevention, Atlanta, GA 30333, USA; ^3^Department of Zoology, Lagos State University, Ojo, Lagos, Nigeria

## Abstract

A study was conducted to detect and identify enteric microsporidian species in 43 children from Oyo state, Nigeria. Using nested polymerase chain reaction, 9.3% of the children were identified as positive for *Enterocytozoon bieneusi*. DNA sequencing of the PCR products showed the presence of three known genotypes (two isolates of genotype D and one of genotype K) and one new genotype. This study suggests that either human or animal (or both) could be the infection source for the children, since identified genotypes D and K have been previously detected in both immunocompromised and immunocompetent patients and domestic animals. The identification of high diversity also suggests intensive transmission of microsporidiosis in the studied area.

## 1. Introduction

Microsporidia are enteric protozoan parasites that are increasingly recognized as important pathogens in immunocompromised persons and malnourished children. Some of the species of this organism infecting humans have been found in animals and water, thereby raising concerns for zoonotic, foodborne, and waterborne transmission [1–3]. *Enterocytozoon bieneusi* is the most common microsporidian species in humans, infecting enterocytes and other epithelial cells of human [1]. Although given lesser attention, the parasite is emerging as an important etiology of diarrhoea in immunocompromised and immunocompetent persons [1]. *Enterocytozoon bieneusi* causes chronic diarrhea and weight loss in human-immunodeficiency-virus- (HIV-) infected patients, and the infection is increasingly observed in organ transplant recipients [4–6].

While there are a lot of studies on opportunistic organisms like *Cryptosporidium *and *Giardia*, microsporidiosis is rarely studied. This preliminary study was conducted to identify the species- and genotypes-involved microsporidiosis in children from Oyo state of Nigeria.

## 2. Materials and Methods

Fecal samples were collected randomly from 43 (33 (76.7%) diarrhoeic and 10 (23.3%) nondiarrhoeic) children attending an outpatient department of a hospital in Oyo state, southwestern Nigeria. These samples were processed for DNA extraction with FastDNA Spin Kit for soil (MP Biologicals, Solon, Ohio, USA). For the detection of *E. bieneusi*, a nested PCR was used to amplify a ~400-bp fragment of the internal transcribed spacer (ITS) region of the rRNA gene [7]. The PCR products were visualized on 1% agarose gel after ethidium bromide staining [7]. *Enterocytozoon bieneusi* was genotyped by sequence analysis of the ITS PCR products.

DNA sequencing of *E. bieneusi* ITS product was done using an ABI Prism 3130 Genetic Analyzer and the BigDye Terminator Sequencing Kit (Applied Biosystems, Foster City, Calif, USA). The nucleotide sequences obtained were aligned with each other and reference sequences downloaded from the GenBank using ClustalX (http://www.clustal.org/). *E. bieneusi* genotypes were determined by their similarity to the sequences from known genotypes and subtypes. The established terminology was used in naming the *E. bieneusi* genotypes [1].

## 3. Results and Discussion

PCR analysis of the ITS gene identified *E. bieneusi* in four samples (Table 1). DNA sequencing of the PCR products identified the presence of three genotypes. Two of the genotypes were known ones, D (in two isolates) and K (in one isolate), and one had two nucleotide substitutions comparing to genotype K (in one isolate). Among the four *E. bieneusi*-positive children, three had diarrhoea and one did not have diarrhoea (Table 1). 

While in this pilot study, only a small number of children were found positive for *E. bieneusi*, the genotype and subtype diversity of the parasite was high, suggesting that there was intensive transmission of microsporidiosis in this area. The source of *E. bieneusi* in the studied children is not known. However, both genotypes D and K identified in the study have been previously detected in both immunocompromised and immunocompetent patients and domestic animals. [1] While, these genotypes could be of animal origin, a data from *Cryptosporidium* investigation conducted on the same group of patient [8] suggests a strong possibility of anthroponotic transmission of these two *E. bieneusi* genotypes in the study area. More systematic collection of epidemiological data and sampling of animals, drinking water, and fresh produce are needed to clarify the source of *E. bieneusi* in Nigerian children.

Geographically isolated *E. bieneusi* genotypes/subtypes are probably circulating in Nigerian children, and even one of the *E. bieneusi* genotypes represents a new genotype.

In conclusion, our study reports high genotype and subtype diversity of *E. bieneusi* and also suggests that anthroponotic transmissions may play a significant role in the epidemiology of microsporidiosis. More studies are needed on the source and transmission of microsporidiosis in Nigeria and in other developing countries.

## Figures and Tables

**Table 1:** Showing the prevalence of *Enterocytozoon bieneusi* isolates from Nigerian children.

Age (months)	Sample size	Diarrhoeic	Nondiarrhoeic	No positive^a^ (%)	Genotype
1–6	10	9	1	1 (2.3)	New genotype* (1)
6–12	22	18	4	2 (4.7)	Genotype D (1) Genotype D (1)
>12	11	7	4	1 (2.3)	Genotype K (1)

Total	43	34	9	4 (9.3)	

^a^ *E. bieneusi* was detected in 3 and 1 diarrhoeic and nondiarrhoeic persons, respectively. There was no association between the infection and diarrhoea (*P* > 0.05, *χ* ^2^).

*Similar to genotype K (with two nucleotide substitutions).
